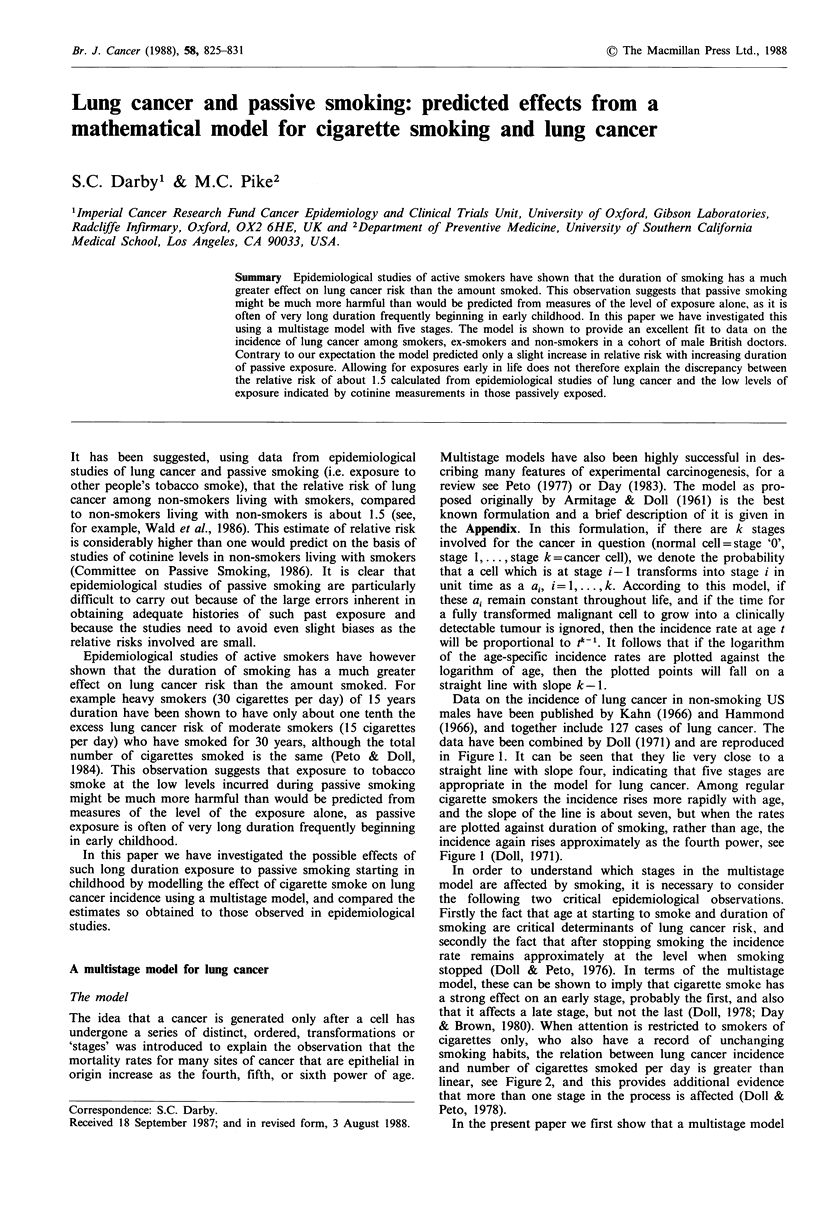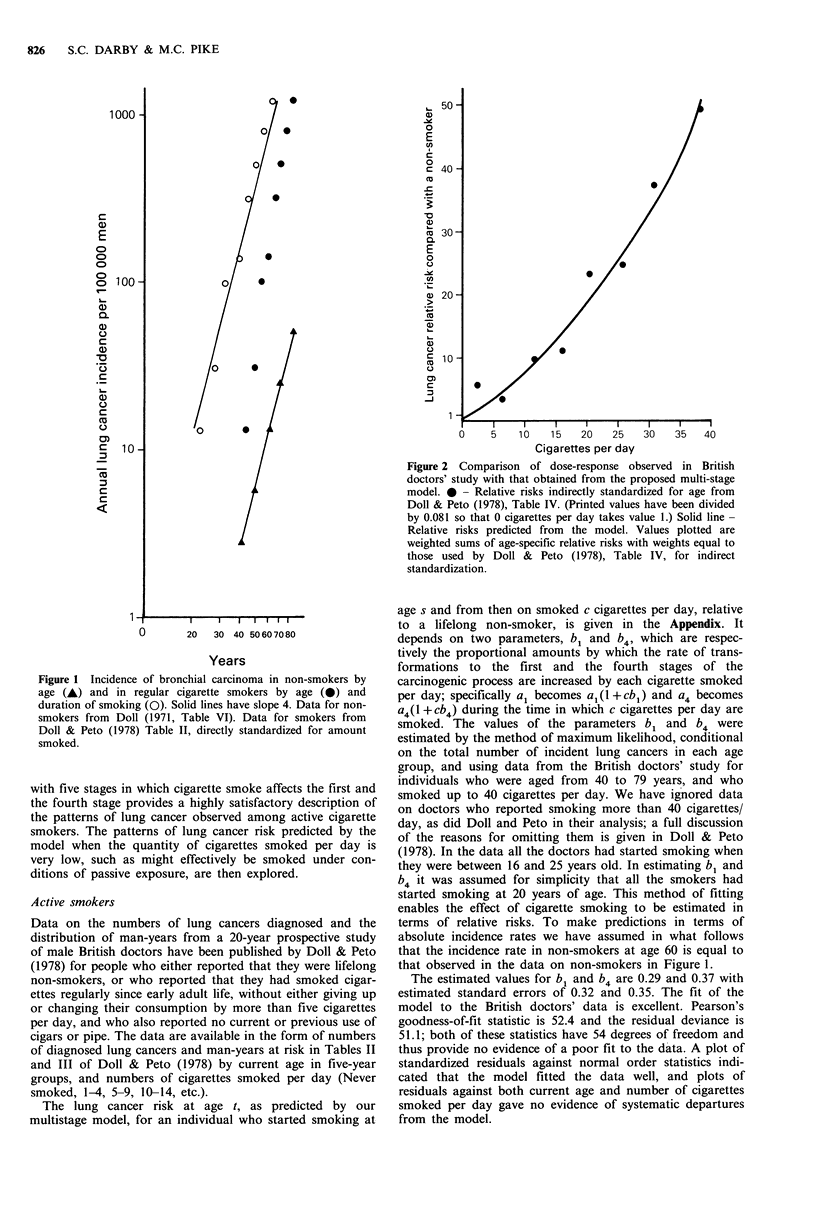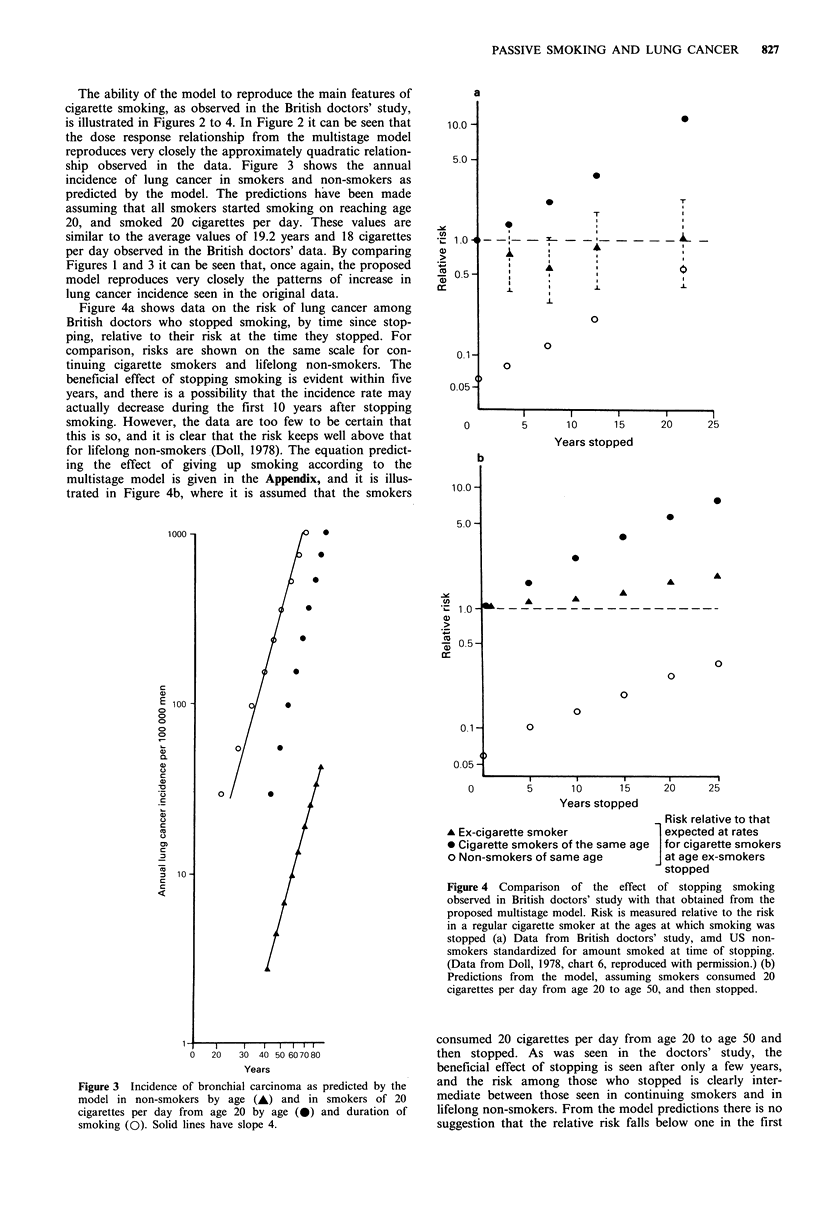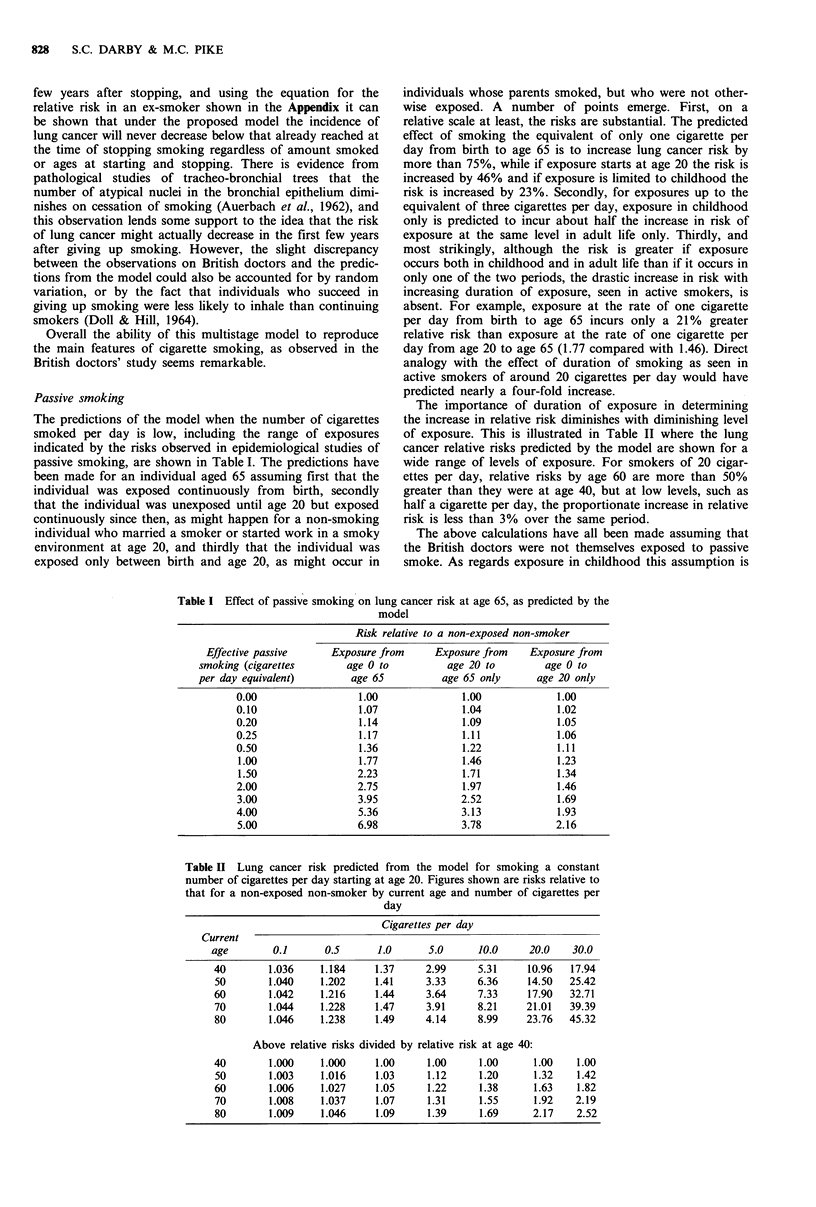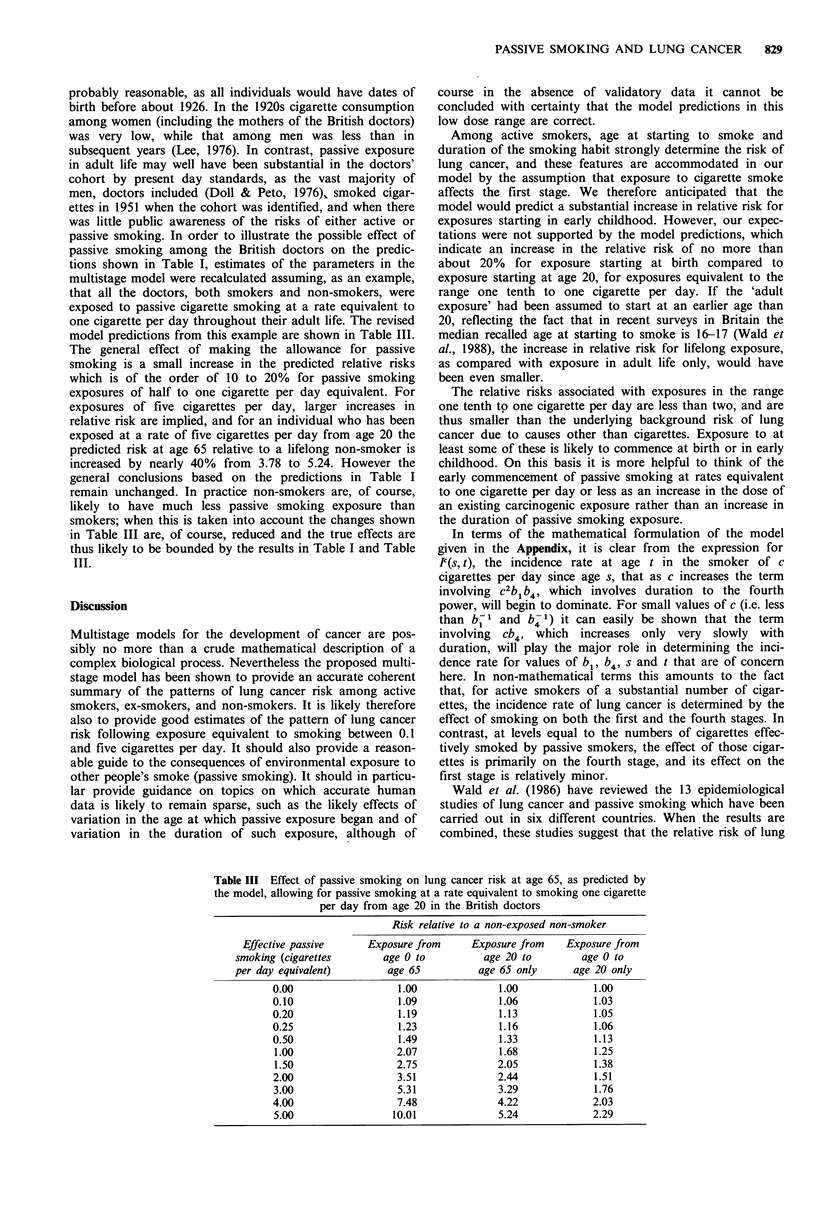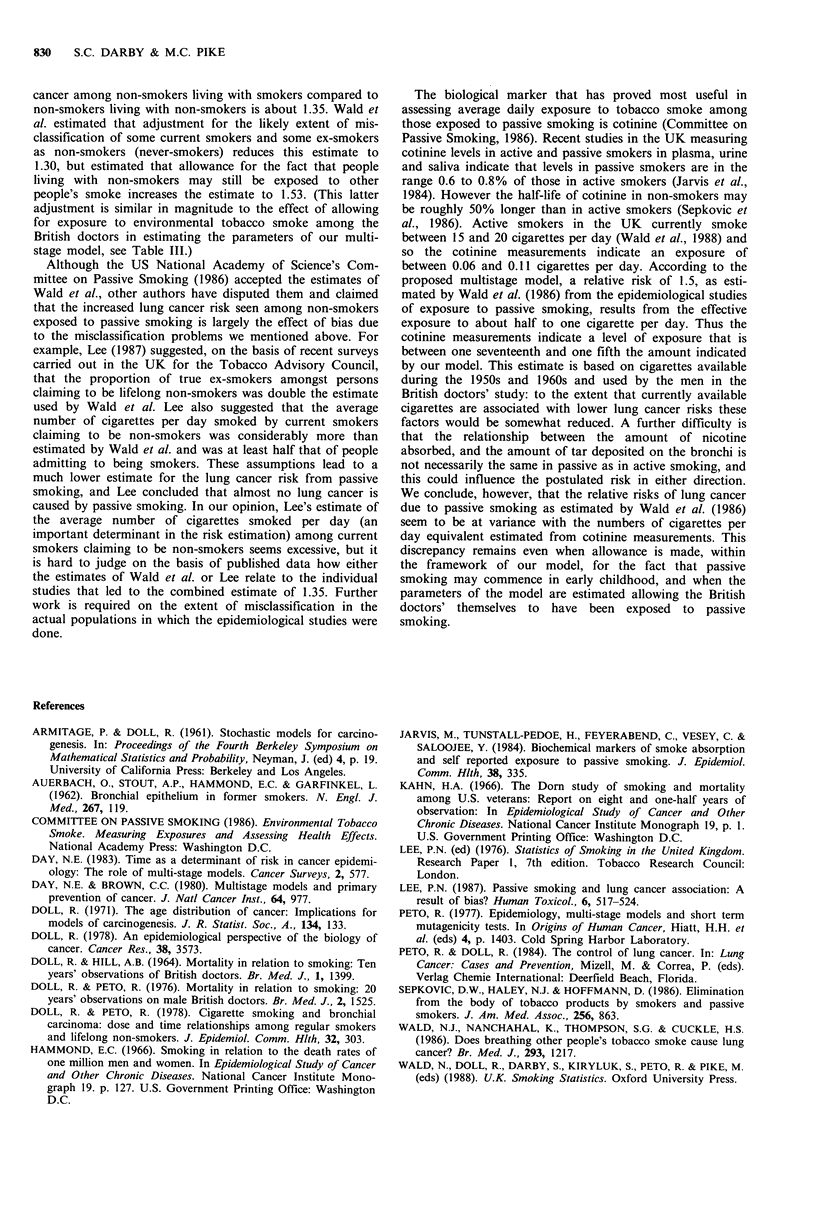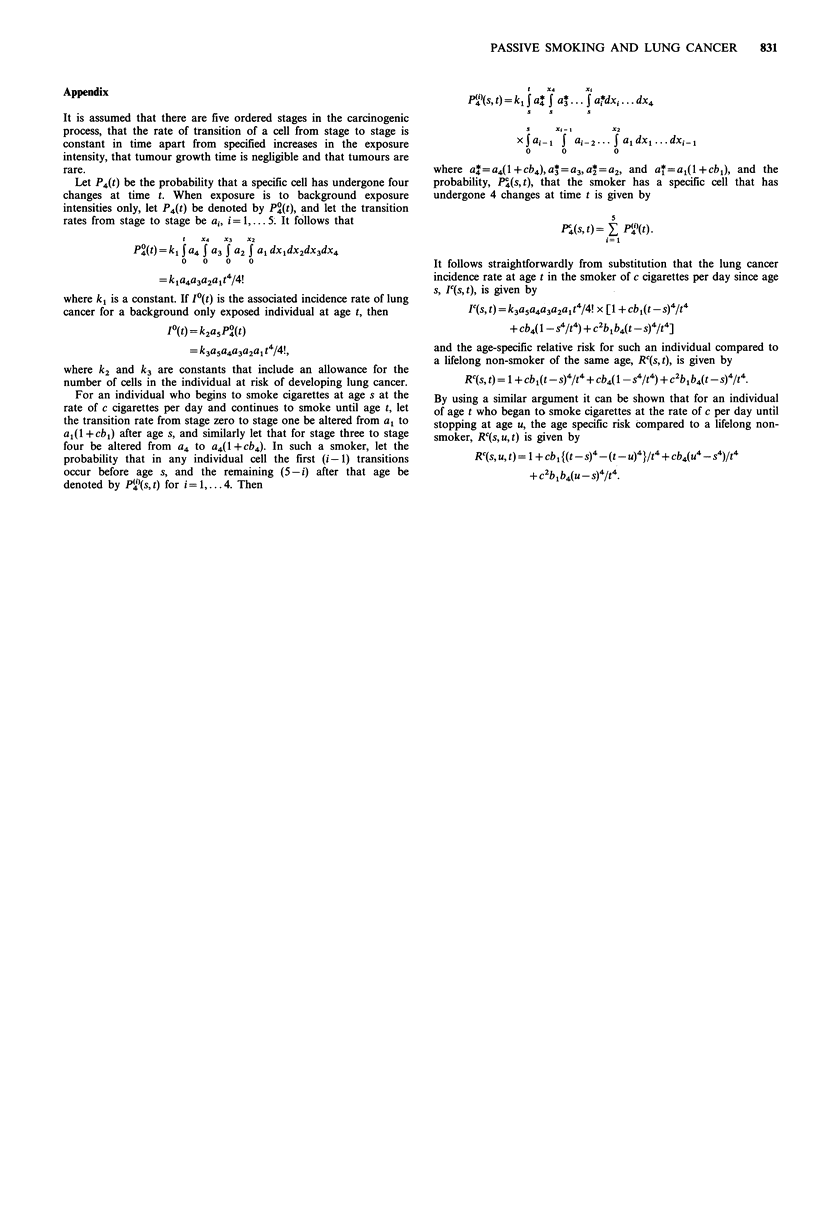# Lung cancer and passive smoking: predicted effects from a mathematical model for cigarette smoking and lung cancer.

**DOI:** 10.1038/bjc.1988.319

**Published:** 1988-12

**Authors:** S. C. Darby, M. C. Pike

**Affiliations:** Imperial Cancer Research Fund Cancer Epidemiology and Clinical Trials Unit, University of Oxford, Gibson Laboratories, Radcliffe Infirmary, Oxford, UK.

## Abstract

Epidemiological studies of active smokers have shown that the duration of smoking has a much greater effect on lung cancer risk than the amount smoked. This observation suggests that passive smoking might be much more harmful than would be predicted from measures of the level of exposure alone, as it is often of very long duration frequently beginning in early childhood. In this paper we have investigated this using a multistage model with five stages. The model is shown to provide an excellent fit to data on the incidence of lung cancer among smokers, ex-smokers and non-smokers in a cohort of male British doctors. Contrary to our expectation the model predicted only a slight increase in relative risk with increasing duration of passive exposure. Allowing for exposures early in life does not therefore explain the discrepancy between the relative risk of about 1.5 calculated from epidemiological studies of lung cancer and the low levels of exposure indicated by cotinine measurements in those passively exposed.


Br. J. Cancer (1988), 58, 825–831 © The Macmillan Press Ltd., 1988

### Lung cancer and passive smoking: predicted effects from a mathematical model for cigarette smoking and lung cancer

S.C. Darby[1] & M.C. Pike[2]

[1]Imperial Cancer Research Fund Cancer Epidemiology and Clinical Trials Unit, University of Oxford, Gibson Laboratories, Radcliffe Infirmary, Oxford, OX2 6HE, UK and [2]Department of Preventive Medicine, University of Southern California Medical School, Los Angeles, CA 90033, USA.

**Summary** Epidemiological studies of active smokers have shown that the duration of smoking has a much greater effect on lung cancer risk than the amount smoked. This observation suggests that passive smoking might be much more harmful than would be predicted from measures of the level of exposure alone, as it is often of very long duration frequently beginning in early childhood. In this paper we have investigated this using a multistage model with five stages. The model is shown to provide an excellent fit to data on the incidence of lung cancer among smokers, ex-smokers and non-smokers in a cohort of male British doctors. Contrary to our expectation the model predicted only a slight increase in relative risk with increasing duration of passive exposure. Allowing for exposures early in life does not therefore explain the discrepancy between the relative risk of about 1.5 calculated from epidemiological studies of lung cancer and the low levels of exposure indicated by cotinine measurements in those passively exposed.

It has been suggested, using data from epidemiological studies of lung cancer and passive smoking (i.e. exposure to other people's tobacco smoke), that the relative risk of lung cancer among non-smokers living with smokers, compared to non-smokers living with non-smokers is about 1.5 (see, for example, Wald *et al.*, 1986). This estimate of relative risk is considerably higher than one would predict on the basis of studies of cotinine levels in non-smokers living with smokers (Committee on Passive Smoking, 1986). It is clear that epidemiological studies of passive smoking are particularly difficult to carry out because of the large errors inherent in obtaining adequate histories of such past exposure and because the studies need to avoid even slight biases as the relative risks involved are small.

Epidemiological studies of active smokers have however shown that the duration of smoking has a much greater effect on lung cancer risk than the amount smoked. For example heavy smokers (30 cigarettes per day) of 15 years duration have been shown to have only about one tenth the excess lung cancer risk of moderate smokers (15 cigarettes per day) who have smoked for 30 years, although the total number of cigarettes smoked is the same (Peto & Doll, 1984). This observation suggests that exposure to tobacco smoke at the low levels incurred during passive smoking might be much more harmful than would be predicted from measures of the level of the exposure alone, as passive exposure is often of very long duration frequently beginning in early childhood.

In this paper we have investigated the possible effects of such long duration exposure to passive smoking starting in childhood by modelling the effect of cigarette smoke on lung cancer incidence using a multistage model, and compared the estimates so obtained to those observed in epidemiological studies.

### A multistage model for lung cancer

### *The model*

The idea that a cancer is generated only after a cell has undergone a series of distinct, ordered, transformations or 'stages' was introduced to explain the observation that the mortality rates for many sites of cancer that are epithelial in origin increase as the fourth, fifth, or sixth power of age.

Correspondence: S.C. Darby.
Received 18 September 1987; and in revised form, 3 August 1988.

Multistage models have also been highly successful in describing many features of experimental carcinogenesis, for a review see Peto (1977) or Day (1983). The model as proposed originally by Armitage & Doll (1961) is the best known formulation and a brief description of it is given in the **Appendix**. In this formulation, if there are $k$ stages involved for the cancer in question (normal cell = stage '0', stage 1, ..., stage $k$ = cancer cell), we denote the probability that a cell which is at stage $i-1$ transforms into stage $i$ in unit time as a $a_i$, $i=1,\ldots,k$. According to this model, if these $a_i$ remain constant throughout life, and if the time for a fully transformed malignant cell to grow into a clinically detectable tumour is ignored, then the incidence rate at age $t$ will be proportional to $t^{k-1}$. It follows that if the logarithm of the age-specific incidence rates are plotted against the logarithm of age, then the plotted points will fall on a straight line with slope $k-1$.

Data on the incidence of lung cancer in non-smoking US males have been published by Kahn (1966) and Hammond (1966), and together include 127 cases of lung cancer. The data have been combined by Doll (1971) and are reproduced in Figure 1. It can be seen that they lie very close to a straight line with slope four, indicating that five stages are appropriate in the model for lung cancer. Among regular cigarette smokers the incidence rises more rapidly with age, and the slope of the line is about seven, but when the rates are plotted against duration of smoking, rather than age, the incidence again rises approximately as the fourth power, see Figure 1 (Doll, 1971).

In order to understand which stages in the multistage model are affected by smoking, it is necessary to consider the following two critical epidemiological observations. Firstly the fact that age at starting to smoke and duration of smoking are critical determinants of lung cancer risk, and secondly the fact that after stopping smoking the incidence rate remains approximately at the level when smoking stopped (Doll & Peto, 1976). In terms of the multistage model, these can be shown to imply that cigarette smoke has a strong effect on an early stage, probably the first, and also that it affects a late stage, but not the last (Doll, 1978; Day & Brown, 1980). When attention is restricted to smokers of cigarettes only, who also have a record of unchanging smoking habits, the relation between lung cancer incidence and number of cigarettes smoked per day is greater than linear, see Figure 2, and this provides additional evidence that more than one stage in the process is affected (Doll & Peto, 1978).

In the present paper we first show that a multistage model

**Figure 1** Incidence of bronchial carcinoma in non-smokers by age (▲) and in regular cigarette smokers by age (●) and duration of smoking (○). Solid lines have slope 4. Data for non-smokers from Doll (1971, Table VI). Data for smokers from Doll & Peto (1978) Table II, directly standardized for amount smoked.

**Figure 2** Comparison of dose-response observed in British doctors' study with that obtained from the proposed multi-stage model. ● – Relative risks indirectly standardized for age from Doll & Peto (1978), Table IV. (Printed values have been divided by 0.081 so that 0 cigarettes per day takes value 1.) Solid line – Relative risks predicted from the model. Values plotted are weighted sums of age-specific relative risks with weights equal to those used by Doll & Peto (1978), Table IV, for indirect standardization.

with five stages in which cigarette smoke affects the first and the fourth stage provides a highly satisfactory description of the patterns of lung cancer observed among active cigarette smokers. The patterns of lung cancer risk predicted by the model when the quantity of cigarettes smoked per day is very low, such as might effectively be smoked under conditions of passive exposure, are then explored.

*Active smokers*

Data on the numbers of lung cancers diagnosed and the distribution of man-years from a 20-year prospective study of male British doctors have been published by Doll & Peto (1978) for people who either reported that they were lifelong non-smokers, or who reported that they had smoked cigarettes regularly since early adult life, without either giving up or changing their consumption by more than five cigarettes per day, and who also reported no current or previous use of cigars or pipe. The data are available in the form of numbers of diagnosed lung cancers and man-years at risk in Tables II and III of Doll & Peto (1978) by current age in five-year groups, and numbers of cigarettes smoked per day (Never smoked, 1–4, 5–9, 10–14, etc.).

The lung cancer risk at age $t$, as predicted by our multistage model, for an individual who started smoking at age $s$ and from then on smoked $c$ cigarettes per day, relative to a lifelong non-smoker, is given in the **Appendix**. It depends on two parameters, $b_1$ and $b_4$, which are respectively the proportional amounts by which the rate of transformations to the first and the fourth stages of the carcinogenic process are increased by each cigarette smoked per day; specifically $a_1$ becomes $a_1(1+cb_1)$ and $a_4$ becomes $a_4(1+cb_4)$ during the time in which $c$ cigarettes per day are smoked. The values of the parameters $b_1$ and $b_4$ were estimated by the method of maximum likelihood, conditional on the total number of incident lung cancers in each age group, and using data from the British doctors' study for individuals who were aged from 40 to 79 years, and who smoked up to 40 cigarettes per day. We have ignored data on doctors who reported smoking more than 40 cigarettes/day, as did Doll and Peto in their analysis; a full discussion of the reasons for omitting them is given in Doll & Peto (1978). In the data all the doctors had started smoking when they were between 16 and 25 years old. In estimating $b_1$ and $b_4$ it was assumed for simplicity that all the smokers had started smoking at 20 years of age. This method of fitting enables the effect of cigarette smoking to be estimated in terms of relative risks. To make predictions in terms of absolute incidence rates we have assumed in what follows that the incidence rate in non-smokers at age 60 is equal to that observed in the data on non-smokers in Figure 1.

The estimated values for $b_1$ and $b_4$ are 0.29 and 0.37 with estimated standard errors of 0.32 and 0.35. The fit of the model to the British doctors' data is excellent. Pearson's goodness-of-fit statistic is 52.4 and the residual deviance is 51.1; both of these statistics have 54 degrees of freedom and thus provide no evidence of a poor fit to the data. A plot of standardized residuals against normal order statistics indicated that the model fitted the data well, and plots of residuals against both current age and number of cigarettes smoked per day gave no evidence of systematic departures from the model.

The ability of the model to reproduce the main features of cigarette smoking, as observed in the British doctors' study, is illustrated in Figures 2 to 4. In Figure 2 it can be seen that the dose response relationship from the multistage model reproduces very closely the approximately quadratic relationship observed in the data. Figure 3 shows the annual incidence of lung cancer in smokers and non-smokers as predicted by the model. The predictions have been made assuming that all smokers started smoking on reaching age 20, and smoked 20 cigarettes per day. These values are similar to the average values of 19.2 years and 18 cigarettes per day observed in the British doctors' data. By comparing Figures 1 and 3 it can be seen that, once again, the proposed model reproduces very closely the patterns of increase in lung cancer incidence seen in the original data.

Figure 4a shows data on the risk of lung cancer among British doctors who stopped smoking, by time since stopping, relative to their risk at the time they stopped. For comparison, risks are shown on the same scale for continuing cigarette smokers and lifelong non-smokers. The beneficial effect of stopping smoking is evident within five years, and there is a possibility that the incidence rate may actually decrease during the first 10 years after stopping smoking. However, the data are too few to be certain that this is so, and it is clear that the risk keeps well above that for lifelong non-smokers (Doll, 1978). The equation predicting the effect of giving up smoking according to the multistage model is given in the **Appendix**, and it is illustrated in Figure 4b, where it is assumed that the smokers consumed 20 cigarettes per day from age 20 to age 50 and then stopped. As was seen in the doctors' study, the beneficial effect of stopping is seen after only a few years, and the risk among those who stopped is clearly intermediate between those seen in continuing smokers and in lifelong non-smokers. From the model predictions there is no suggestion that the relative risk falls below one in the first

**Figure 3** Incidence of bronchial carcinoma as predicted by the model in non-smokers by age (▲) and in smokers of 20 cigarettes per day from age 20 by age (●) and duration of smoking (○). Solid lines have slope 4.

**Figure 4** Comparison of the effect of stopping smoking observed in British doctors' study with that obtained from the proposed multistage model. Risk is measured relative to the risk in a regular cigarette smoker at the ages at which smoking was stopped (a) Data from British doctors' study, amd US non-smokers standardized for amount smoked at time of stopping. (Data from Doll, 1978, chart 6, reproduced with permission.) (b) Predictions from the model, assuming smokers consumed 20 cigarettes per day from age 20 to age 50, and then stopped.

few years after stopping, and using the equation for the relative risk in an ex-smoker shown in the **Appendix** it can be shown that under the proposed model the incidence of lung cancer will never decrease below that already reached at the time of stopping smoking regardless of amount smoked or ages at starting and stopping. There is evidence from pathological studies of tracheo-bronchial trees that the number of atypical nuclei in the bronchial epithelium diminishes on cessation of smoking (Auerbach *et al.*, 1962), and this observation lends some support to the idea that the risk of lung cancer might actually decrease in the first few years after giving up smoking. However, the slight discrepancy between the observations on British doctors and the predictions from the model could also be accounted for by random variation, or by the fact that individuals who succeed in giving up smoking were less likely to inhale than continuing smokers (Doll & Hill, 1964).

Overall the ability of this multistage model to reproduce the main features of cigarette smoking, as observed in the British doctors' study seems remarkable.

*Passive smoking*

The predictions of the model when the number of cigarettes smoked per day is low, including the range of exposures indicated by the risks observed in epidemiological studies of passive smoking, are shown in Table I. The predictions have been made for an individual aged 65 assuming first that the individual was exposed continuously from birth, secondly that the individual was unexposed until age 20 but exposed continuously since then, as might happen for a non-smoking individual who married a smoker or started work in a smoky environment at age 20, and thirdly that the individual was exposed only between birth and age 20, as might occur in individuals whose parents smoked, but who were not otherwise exposed. A number of points emerge. First, on a relative scale at least, the risks are substantial. The predicted effect of smoking the equivalent of only one cigarette per day from birth to age 65 is to increase lung cancer risk by more than 75%, while if exposure starts at age 20 the risk is increased by 46% and if exposure is limited to childhood the risk is increased by 23%. Secondly, for exposures up to the equivalent of three cigarettes per day, exposure in childhood only is predicted to incur about half the increase in risk of exposure at the same level in adult life only. Thirdly, and most strikingly, although the risk is greater if exposure occurs both in childhood and in adult life than if it occurs in only one of the two periods, the drastic increase in risk with increasing duration of exposure, seen in active smokers, is absent. For example, exposure at the rate of one cigarette per day from birth to age 65 incurs only a 21% greater relative risk than exposure at the rate of one cigarette per day from age 20 to age 65 (1.77 compared with 1.46). Direct analogy with the effect of duration of smoking as seen in active smokers of around 20 cigarettes per day would have predicted nearly a four-fold increase.

The importance of duration of exposure in determining the increase in relative risk diminishes with diminishing level of exposure. This is illustrated in Table II where the lung cancer relative risks predicted by the model are shown for a wide range of levels of exposure. For smokers of 20 cigarettes per day, relative risks by age 60 are more than 50% greater than they were at age 40, but at low levels, such as half a cigarette per day, the proportionate increase in relative risk is less than 3% over the same period.

The above calculations have all been made assuming that the British doctors were not themselves exposed to passive smoke. As regards exposure in childhood this assumption is

**Table I** Effect of passive smoking on lung cancer risk at age 65, as predicted by the model

| Effective passive smoking (cigarettes per day equivalent) | Risk relative to a non-exposed non-smoker: Exposure from age 0 to age 65 | Exposure from age 20 to age 65 only | Exposure from age 0 to age 20 only |
|---|---|---|---|
| 0.00 | 1.00 | 1.00 | 1.00 |
| 0.10 | 1.07 | 1.04 | 1.02 |
| 0.20 | 1.14 | 1.09 | 1.05 |
| 0.25 | 1.17 | 1.11 | 1.06 |
| 0.50 | 1.36 | 1.22 | 1.11 |
| 1.00 | 1.77 | 1.46 | 1.23 |
| 1.50 | 2.23 | 1.71 | 1.34 |
| 2.00 | 2.75 | 1.97 | 1.46 |
| 3.00 | 3.95 | 2.52 | 1.69 |
| 4.00 | 5.36 | 3.13 | 1.93 |
| 5.00 | 6.98 | 3.78 | 2.16 |

**Table II** Lung cancer risk predicted from the model for smoking a constant number of cigarettes per day starting at age 20. Figures shown are risks relative to that for a non-exposed non-smoker by current age and number of cigarettes per day

| Current age | Cigarettes per day: 0.1 | 0.5 | 1.0 | 5.0 | 10.0 | 20.0 | 30.0 |
|---|---|---|---|---|---|---|---|
| 40 | 1.036 | 1.184 | 1.37 | 2.99 | 5.31 | 10.96 | 17.94 |
| 50 | 1.040 | 1.202 | 1.41 | 3.33 | 6.36 | 14.50 | 25.42 |
| 60 | 1.042 | 1.216 | 1.44 | 3.64 | 7.33 | 17.90 | 32.71 |
| 70 | 1.044 | 1.228 | 1.47 | 3.91 | 8.21 | 21.01 | 39.39 |
| 80 | 1.046 | 1.238 | 1.49 | 4.14 | 8.99 | 23.76 | 45.32 |
| | Above relative risks divided by relative risk at age 40: | | | | | | |
| 40 | 1.000 | 1.000 | 1.00 | 1.00 | 1.00 | 1.00 | 1.00 |
| 50 | 1.003 | 1.016 | 1.03 | 1.12 | 1.20 | 1.32 | 1.42 |
| 60 | 1.006 | 1.027 | 1.05 | 1.22 | 1.38 | 1.63 | 1.82 |
| 70 | 1.008 | 1.037 | 1.07 | 1.31 | 1.55 | 1.92 | 2.19 |
| 80 | 1.009 | 1.046 | 1.09 | 1.39 | 1.69 | 2.17 | 2.52 |

probably reasonable, as all individuals would have dates of birth before about 1926. In the 1920s cigarette consumption among women (including the mothers of the British doctors) was very low, while that among men was less than in subsequent years (Lee, 1976). In contrast, passive exposure in adult life may well have been substantial in the doctors' cohort by present day standards, as the vast majority of men, doctors included (Doll & Peto, 1976), smoked cigarettes in 1951 when the cohort was identified, and when there was little public awareness of the risks of either active or passive smoking. In order to illustrate the possible effect of passive smoking among the British doctors on the predictions shown in Table I, estimates of the parameters in the multistage model were recalculated assuming, as an example, that all the doctors, both smokers and non-smokers, were exposed to passive cigarette smoking at a rate equivalent to one cigarette per day throughout their adult life. The revised model predictions from this example are shown in Table III. The general effect of making the allowance for passive smoking is a small increase in the predicted relative risks which is of the order of 10 to 20% for passive smoking exposures of half to one cigarette per day equivalent. For exposures of five cigarettes per day, larger increases in relative risk are implied, and for an individual who has been exposed at a rate of five cigarettes per day from age 20 the predicted risk at age 65 relative to a lifelong non-smoker is increased by nearly 40% from 3.78 to 5.24. However the general conclusions based on the predictions in Table I remain unchanged. In practice non-smokers are, of course, likely to have much less passive smoking exposure than smokers; when this is taken into account the changes shown in Table III are, of course, reduced and the true effects are thus likely to be bounded by the results in Table I and Table III.

### Discussion

Multistage models for the development of cancer are possibly no more than a crude mathematical description of a complex biological process. Nevertheless the proposed multistage model has been shown to provide an accurate coherent summary of the patterns of lung cancer risk among active smokers, ex-smokers, and non-smokers. It is likely therefore also to provide good estimates of the pattern of lung cancer risk following exposure equivalent to smoking between 0.1 and five cigarettes per day. It should also provide a reasonable guide to the consequences of environmental exposure to other people's smoke (passive smoking). It should in particular provide guidance on topics on which accurate human data is likely to remain sparse, such as the likely effects of variation in the age at which passive exposure began and of variation in the duration of such exposure, although of course in the absence of validatory data it cannot be concluded with certainty that the model predictions in this low dose range are correct.

Among active smokers, age at starting to smoke and duration of the smoking habit strongly determine the risk of lung cancer, and these features are accommodated in our model by the assumption that exposure to cigarette smoke affects the first stage. We therefore anticipated that the model would predict a substantial increase in relative risk for exposures starting in early childhood. However, our expectations were not supported by the model predictions, which indicate an increase in the relative risk of no more than about 20% for exposure starting at birth compared to exposure starting at age 20, for exposures equivalent to the range one tenth to one cigarette per day. If the 'adult exposure' had been assumed to start at an earlier age than 20, reflecting the fact that in recent surveys in Britain the median recalled age at starting to smoke is 16–17 (Wald *et al.*, 1988), the increase in relative risk for lifelong exposure, as compared with exposure in adult life only, would have been even smaller.

The relative risks associated with exposures in the range one tenth to one cigarette per day are less than two, and are thus smaller than the underlying background risk of lung cancer due to causes other than cigarettes. Exposure to at least some of these is likely to commence at birth or in early childhood. On this basis it is more helpful to think of the early commencement of passive smoking at rates equivalent to one cigarette per day or less as an increase in the dose of an existing carcinogenic exposure rather than an increase in the duration of passive smoking exposure.

In terms of the mathematical formulation of the model given in the **Appendix**, it is clear from the expression for $I^c(s,t)$, the incidence rate at age $t$ in the smoker of $c$ cigarettes per day since age $s$, that as $c$ increases the term involving $c^2b_1b_4$, which involves duration to the fourth power, will begin to dominate. For small values of $c$ (i.e. less than $b_1^{-1}$ and $b_4^{-1}$) it can easily be shown that the term involving $cb_4$, which increases only very slowly with duration, will play the major role in determining the incidence rate for values of $b_1$, $b_4$, $s$ and $t$ that are of concern here. In non-mathematical terms this amounts to the fact that, for active smokers of a substantial number of cigarettes, the incidence rate of lung cancer is determined by the effect of smoking on both the first and the fourth stages. In contrast, at levels equal to the numbers of cigarettes effectively smoked by passive smokers, the effect of those cigarettes is primarily on the fourth stage, and its effect on the first stage is relatively minor.

Wald *et al.* (1986) have reviewed the 13 epidemiological studies of lung cancer and passive smoking which have been carried out in six different countries. When the results are combined, these studies suggest that the relative risk of lung

**Table III** Effect of passive smoking on lung cancer risk at age 65, as predicted by the model, allowing for passive smoking at a rate equivalent to smoking one cigarette per day from age 20 in the British doctors

| | *Risk relative to a non-exposed non-smoker* | | |
|---|---|---|---|
| *Effective passive smoking (cigarettes per day equivalent)* | *Exposure from age 0 to age 65* | *Exposure from age 20 to age 65 only* | *Exposure from age 0 to age 20 only* |
| 0.00 | 1.00 | 1.00 | 1.00 |
| 0.10 | 1.09 | 1.06 | 1.03 |
| 0.20 | 1.19 | 1.13 | 1.05 |
| 0.25 | 1.23 | 1.16 | 1.06 |
| 0.50 | 1.49 | 1.33 | 1.13 |
| 1.00 | 2.07 | 1.68 | 1.25 |
| 1.50 | 2.75 | 2.05 | 1.38 |
| 2.00 | 3.51 | 2.44 | 1.51 |
| 3.00 | 5.31 | 3.29 | 1.76 |
| 4.00 | 7.48 | 4.22 | 2.03 |
| 5.00 | 10.01 | 5.24 | 2.29 |

cancer among non-smokers living with smokers compared to non-smokers living with non-smokers is about 1.35. Wald *et al.* estimated that adjustment for the likely extent of misclassification of some current smokers and some ex-smokers as non-smokers (never-smokers) reduces this estimate to 1.30, but estimated that allowance for the fact that people living with non-smokers may still be exposed to other people's smoke increases the estimate to 1.53. (This latter adjustment is similar in magnitude to the effect of allowing for exposure to environmental tobacco smoke among the British doctors in estimating the parameters of our multistage model, see Table III.)

Although the US National Academy of Science's Committee on Passive Smoking (1986) accepted the estimates of Wald *et al.*, other authors have disputed them and claimed that the increased lung cancer risk seen among non-smokers exposed to passive smoking is largely the effect of bias due to the misclassification problems we mentioned above. For example, Lee (1987) suggested, on the basis of recent surveys carried out in the UK for the Tobacco Advisory Council, that the proportion of true ex-smokers amongst persons claiming to be lifelong non-smokers was double the estimate used by Wald *et al.* Lee also suggested that the average number of cigarettes per day smoked by current smokers claiming to be non-smokers was considerably more than estimated by Wald *et al.* and was at least half that of people admitting to being smokers. These assumptions lead to a much lower estimate for the lung cancer risk from passive smoking, and Lee concluded that almost no lung cancer is caused by passive smoking. In our opinion, Lee's estimate of the average number of cigarettes smoked per day (an important determinant in the risk estimation) among current smokers claiming to be non-smokers seems excessive, but it is hard to judge on the basis of published data how either the estimates of Wald *et al.* or Lee relate to the individual studies that led to the combined estimate of 1.35. Further work is required on the extent of misclassification in the actual populations in which the epidemiological studies were done.

The biological marker that has proved most useful in assessing average daily exposure to tobacco smoke among those exposed to passive smoking is cotinine (Committee on Passive Smoking, 1986). Recent studies in the UK measuring cotinine levels in active and passive smokers in plasma, urine and saliva indicate that levels in passive smokers are in the range 0.6 to 0.8% of those in active smokers (Jarvis *et al.*, 1984). However the half-life of cotinine in non-smokers may be roughly 50% longer than in active smokers (Sepkovic *et al.*, 1986). Active smokers in the UK currently smoke between 15 and 20 cigarettes per day (Wald *et al.*, 1988) and so the cotinine measurements indicate an exposure of between 0.06 and 0.11 cigarettes per day. According to the proposed multistage model, a relative risk of 1.5, as estimated by Wald *et al.* (1986) from the epidemiological studies of exposure to passive smoking, results from the effective exposure to about half to one cigarette per day. Thus the cotinine measurements indicate a level of exposure that is between one seventeenth and one fifth the amount indicated by our model. This estimate is based on cigarettes available during the 1950s and 1960s and used by the men in the British doctors' study: to the extent that currently available cigarettes are associated with lower lung cancer risks these factors would be somewhat reduced. A further difficulty is that the relationship between the amount of nicotine absorbed, and the amount of tar deposited on the bronchi is not necessarily the same in passive as in active smoking, and this could influence the postulated risk in either direction. We conclude, however, that the relative risks of lung cancer due to passive smoking as estimated by Wald *et al.* (1986) seem to be at variance with the numbers of cigarettes per day equivalent estimated from cotinine measurements. This discrepancy remains even when allowance is made, within the framework of our model, for the fact that passive smoking may commence in early childhood, and when the parameters of the model are estimated allowing the British doctors' themselves to have been exposed to passive smoking.

### References

ARMITAGE, P. & DOLL, R. (1961). Stochastic models for carcinogenesis. In: *Proceedings of the Fourth Berkeley Symposium on Mathematical Statistics and Probability*, Neyman, J. (ed) **4**, p. 19. University of California Press: Berkeley and Los Angeles.

AUERBACH, O., STOUT, A.P., HAMMOND, E.C. & GARFINKEL, L. (1962). Bronchial epithelium in former smokers. *N. Engl. J. Med.*, **267**, 119.

COMMITTEE ON PASSIVE SMOKING (1986). *Environmental Tobacco Smoke. Measuring Exposures and Assessing Health Effects*. National Academy Press: Washington D.C.

DAY, N.E. (1983). Time as a determinant of risk in cancer epidemiology: The role of multi-stage models. *Cancer Surveys*, **2**, 577.

DAY, N.E. & BROWN, C.C. (1980). Multistage models and primary prevention of cancer. *J. Natl Cancer Inst.*, **64**, 977.

DOLL, R. (1971). The age distribution of cancer: Implications for models of carcinogenesis. *J. R. Statist. Soc., A.*, **134**, 133.

DOLL, R. (1978). An epidemiological perspective of the biology of cancer. *Cancer Res.*, **38**, 3573.

DOLL, R. & HILL, A.B. (1964). Mortality in relation to smoking: Ten years' observations of British doctors. *Br. Med. J.*, **1**, 1399.

DOLL, R. & PETO, R. (1976). Mortality in relation to smoking: 20 years' observations on male British doctors. *Br. Med. J.*, **2**, 1525.

DOLL, R. & PETO, R. (1978). Cigarette smoking and bronchial carcinoma: dose and time relationships among regular smokers and lifelong non-smokers. *J. Epidemiol. Comm. Hlth*, **32**, 303.

HAMMOND, E.C. (1966). Smoking in relation to the death rates of one million men and women. In *Epidemiological Study of Cancer and Other Chronic Diseases*. National Cancer Institute Monograph 19. p. 127. U.S. Government Printing Office: Washington D.C.

JARVIS, M., TUNSTALL-PEDOE, H., FEYERABEND, C., VESEY, C. & SALOOJEE, Y. (1984). Biochemical markers of smoke absorption and self reported exposure to passive smoking. *J. Epidemiol. Comm. Hlth*, **38**, 335.

KAHN, H.A. (1966). The Dorn study of smoking and mortality among U.S. veterans: Report on eight and one-half years of observation: In *Epidemiological Study of Cancer and Other Chronic Diseases*. National Cancer Institute Monograph 19, p. 1. U.S. Government Printing Office: Washington D.C.

LEE, P.N. (ed) (1976). *Statistics of Smoking in the United Kingdom*. Research Paper 1, 7th edition. Tobacco Research Council: London.

LEE, P.N. (1987). Passive smoking and lung cancer association: A result of bias? *Human Toxicol.*, **6**, 517–524.

PETO, R. (1977). Epidemiology, multi-stage models and short term mutagenicity tests. In *Origins of Human Cancer*, Hiatt, H.H. *et al.* (eds) **4**, p. 1403. Cold Spring Harbor Laboratory.

PETO, R. & DOLL, R. (1984). The control of lung cancer. In: *Lung Cancer: Cases and Prevention*, Mizell, M. & Correa, P. (eds). Verlag Chemie International: Deerfield Beach, Florida.

SEPKOVIC, D.W., HALEY, N.J. & HOFFMANN, D. (1986). Elimination from the body of tobacco products by smokers and passive smokers. *J. Am. Med. Assoc.*, **256**, 863.

WALD, N.J., NANCHAHAL, K., THOMPSON, S.G. & CUCKLE, H.S. (1986). Does breathing other people's tobacco smoke cause lung cancer? *Br. Med. J.*, **293**, 1217.

WALD, N., DOLL, R., DARBY, S., KIRYLUK, S., PETO, R. & PIKE, M. (eds) (1988). *U.K. Smoking Statistics*. Oxford University Press.

**Appendix**

It is assumed that there are five ordered stages in the carcinogenic process, that the rate of transition of a cell from stage to stage is constant in time apart from specified increases in the exposure intensity, that tumour growth time is negligible and that tumours are rare.

Let $P_4(t)$ be the probability that a specific cell has undergone four changes at time $t$. When exposure is to background exposure intensities only, let $P_4(t)$ be denoted by $P_4^0(t)$, and let the transition rates from stage to stage be $a_i$, $i=1,\dots 5$. It follows that

$$P_4^0(t)=k_1\int_0^t a_4\int_0^{x_4} a_3\int_0^{x_3} a_2\int_0^{x_2} a_1\,dx_1dx_2dx_3dx_4$$

$$=k_1a_4a_3a_2a_1t^4/4!$$

where $k_1$ is a constant. If $I^0(t)$ is the associated incidence rate of lung cancer for a background only exposed individual at age $t$, then

$$I^0(t)=k_2a_5P_4^0(t)$$

$$=k_3a_5a_4a_3a_2a_1t^4/4!,$$

where $k_2$ and $k_3$ are constants that include an allowance for the number of cells in the individual at risk of developing lung cancer.

For an individual who begins to smoke cigarettes at age $s$ at the rate of $c$ cigarettes per day and continues to smoke until age $t$, let the transition rate from stage zero to stage one be altered from $a_1$ to $a_1(1+cb_1)$ after age $s$, and similarly let that for stage three to stage four be altered from $a_4$ to $a_4(1+cb_4)$. In such a smoker, let the probability that in any individual cell the first $(i-1)$ transitions occur before age $s$, and the remaining $(5-i)$ after that age be denoted by $P_4^{(i)}(s,t)$ for $i=1,\dots 4$. Then

$$P_4^{(i)}(s,t)=k_1\int_s^t a_4^*\int_s^{x_4} a_3^*\dots\int_s^{x_i} a_i^*dx_i\dots dx_4$$
$$\times\int_0^s a_{i-1}\int_0^{x_{i-1}} a_{i-2}\dots\int_0^{x_2} a_1\,dx_1\dots dx_{i-1}$$

where $a_4^*=a_4(1+cb_4), a_3^*=a_3, a_2^*=a_2$, and $a_1^*=a_1(1+cb_1)$, and the probability, $P_4^c(s,t)$, that the smoker has a specific cell that has undergone 4 changes at time $t$ is given by

$$P_4^c(s,t)=\sum_{i=1}^{5}P_4^{(i)}(t).$$

It follows straightforwardly from substitution that the lung cancer incidence rate at age $t$ in the smoker of $c$ cigarettes per day since age $s$, $I^c(s,t)$, is given by

$$I^c(s,t)=k_3a_5a_4a_3a_2a_1t^4/4!\times[1+cb_1(t-s)^4/t^4$$

$$+cb_4(1-s^4/t^4)+c^2b_1b_4(t-s)^4/t^4]$$

and the age-specific relative risk for such an individual compared to a lifelong non-smoker of the same age, $R^c(s,t)$, is given by

$$R^c(s,t)=1+cb_1(t-s)^4/t^4+cb_4(1-s^4/t^4)+c^2b_1b_4(t-s)^4/t^4.$$

By using a similar argument it can be shown that for an individual of age $t$ who began to smoke cigarettes at the rate of $c$ per day until stopping at age $u$, the age specific risk compared to a lifelong non-smoker, $R^c(s,u,t)$ is given by

$$R^c(s,u,t)=1+cb_1\{(t-s)^4-(t-u)^4\}/t^4+cb_4(u^4-s^4)/t^4$$

$$+c^2b_1b_4(u-s)^4/t^4.$$